# Epidemiology of illnesses encountered during a marathon race – an EMS perspective

**DOI:** 10.1186/1865-1380-8-S1-O3

**Published:** 2015-04-22

**Authors:** Yusra Fatima, Amaan Ahmed, Sohail Abdullah, Imran Shareef

**Affiliations:** 1 Department of Emergency Medicine, CARE hospitals, Hyderabad, India

## Background and objectives

There is a dearth of good data from the Emergency Medical Services (EMS) regarding emergencies during a Marathon race. This study aims to document the various emergencies that can occur during the course of a marathon in India. We hope that this study will enable planners to create a database and to improve emergency preparedness for future such events.

## Methods

### Study population

A total of 10,000 runners in the age group of 18-80 years, participated in the full (42.2 km) marathon, half (21.1 km) marathon and the 5 K run. Both trained and untrained runners took part in the race.

### Location

Southern Indian city of Hyderabad, on the 24^th^ of August from 5:00 AM to 1: 00 PM.

### Race conditions

Urban and semi-urban parts of the city with both paved and un-paved roads, maximum recorded temperature during the race was 35° Celsius.

A prospective descriptive epidemiological analysis of race data of runners presenting with emergencies was done using a standardized patient data collection form given to medical aid providers. Triaging was done according to Canadian triage and acuity scale.

## Results

Of 10,000 runners, 252 runners required medical assistance during the course of the race.

Of all the patients presenting to EMS 248 were Triage priority 3 or 4 requiring urgent or less urgent care

• Exercise related musculoskeletal injuries accounted for 200 (79%) of the total cases

• Dehydration related cramps accounted for 33 (13%) of the cases requiring oral or IV fluid rehydration

• Terrain related blisters and foot injuries – 9 (3.5%) patients

• Vomitings - 4 (1.5%)

A total of 4 patients were triage priority 2 requiring Emergent care – Transported by EMS to the nearest tertiary care hospital.

A 24 year old with symptomatic hypoglycaemia, requiring Dextrose infusion

A 45 year old male with Altered mental status secondary to Heat exhaustion and hypotension requiring IV fluid resuscitation

A 70 year old male with Hypotension and dizziness requiring IV fluid resuscitation

A 23 year old girl who collapsed with hypotension and heat exhaustion requiring IV fluid resuscitation.

## Conclusion

Long-distance running can be a safe and benign sport; however there were serious heat related illnesses requiring hospitalization of runners in this study. A well prepared EMS system is paramount to hosting a marathon in India due to extreme temperatures and mass participation.

## Note

Consent was taken from the four patients who were triaged as priority 2 and received in-hospital care.

